# Metabolic Characterisation of the Midgut of *Bombyx mori* Varieties after BmNPV Infection Using GC-MS-Based Metabolite Profiling

**DOI:** 10.3390/ijms21134707

**Published:** 2020-07-01

**Authors:** Heying Qian, Gang Li, Guodong Zhao, Mingzhu Liu, Anying Xu

**Affiliations:** 1 The Sericultural Research Institute, Jiangsu University of Science and Technology, Zhenjiang 212003, China; gangsri@just.edu.cn (G.L.); sdgdzhao@163.com (G.Z.); lmz-008@163.com (M.L.); 2 The key Laboratory of silkworm and mulberry genetic improvement, Ministry of Agriculture, Chinese Academy of Agricultural Science, Zhenjiang 212018, China

**Keywords:** silkworm, BmNPV, GC-MS, RNA-seq, metabolic pathway

## Abstract

*Bombyx mori* nucleopolyhedrovirus (BmNPV) is a silkworm disease that is especially harmful to cocoon production and seriously restricts sericultural development. Our laboratory successfully cultivated a new highly BmNPV-resistant silkworm variety, *Huakang 2*; however, its mechanism of BmNPV resistance remains unclear. To understand its resistance mechanism, we conducted a metabolomic and transcriptomic study of the midgut of silkworm varieties, Baiyu N and Baiyu after BmNPV infection. We identified 451 differential metabolites, which were mostly comprised of small molecules, such as saccharides, acids, amines, alcohols, and glycosides. We found that the primary differences in disease resistance between the silkworm varieties are metabolic-pathways, tryptophan metabolism, oxidative phosphorylation, ABC-transporters, beta-alanine metabolism, and phenylalanine metabolism. Combined analysis with transcriptomic data suggested that tryptophan metabolism and oxidative phosphorylation are closely related to the silkworms’ BmNPV resistance. We hypothesize that the roles of the two metabolic pathways in the BmNPV resistance mechanism might be the following: Oxidative phosphorylation generates a large amount of adenosine triphosphate (ATP) in response to BmNPV infection to provide silkworms the energy required for establishing BmNPV resistance. Tryptophan metabolism then activates the aryl hydrocarbon receptor (AhR) through the exogenous virus BmNPV, which activates the silkworm’s immune system to defeat BmNPV infections.

## 1. Introduction

The silkworm is an insect with large economic value for humans. According to data compiled by China’s National Bureau of Statistics, there were 12.62 million acres of mulberry fields and more than 30 million households working in the sericulture industry in China in 2012. The annual silkworm cocoon yield reached 688,000 metric tonnes, while the raw silk yield reached 172,200 metric tonnes [1]. However, sericulture continues to be plagued by silkworm diseases; nuclear polyhedrosis is a severe silkworm disease that has prevented sustainable sericulture development. According to Li et al. [2], BmNPV is the most common silkworm disease to afflict and concern silkworm raisers in a survey of 1782 rural households in 91 counties within 14 provinces of China. The disease is caused by BmNPV infection and is extremely difficult to prevent and control. More than 75% of silkworm raisers interviewed by Li et al. have reported losses caused by BmNPV infections. It is widely recognized in the sericulture industry that the best solution for fighting the disease is to cultivate an anti-BmNPV silkworm variety. Therefore, studying the anti-BmNPV immune mechanism of silkworms, analyzing antiviral immune signaling pathways, and screening and identifying antiviral molecules can not only result in the development of disease-resistant varieties, but also improve the theoretical system of insect anti-virus immunity.

Our laboratory discovered a unique germplasm highly resistant to BmNPV [3] while screening silkworm germplasm resources to identify the BmNPV resistant germplasm. Resistance breeding, as an effective way to prevent and control BmNPV infection, is also an important issue requiring urgent attention. We started breeding the anti-BmNPV silkworm variety through hybridization and backcross in 2005. Eight years later, we arrived at a silkworm variety that is highly resistant to BmNPV known as *Huakang 2*. (The silkworm variety, *Huakang 2*, is a cross between Qiufeng N and Baiyu N varieties) [4]. Starting from the first day of the second instar, *Huakang 2* silkworms contained more than 10^9^/mL of LC_50_ value to fight against BmNPV infection, which makes its disease resistance 1000 times higher than that of control silkworm varieties, namely Qiufeng and Baiyu. The *Huakang 2* silkworm variety has been dispersed throughout 45 towns within 15 provinces of China, and it has shown strong BmNPV tolerance. Thus, the popularization of this new silkworm variety has eradicated BmNPV infections, thereby ensuring high cocoon yields in silkworm farms.

The anti-BmNPV mechanism of silkworms has long been important in sericultural studies, and many breakthroughs have been made over the years [5,6,7,8]. After a silkworm ingests the polyhedral BmNPV, alkaline digestive juices secreted from the silkworm’s midgut dissolve the polyhedrin to release the occlusion-derived virus (ODV). The ODV first infects the silkworm’s midgut epithelium before completely infecting the silkworm [9]. Therefore, the midgut is the first barrier against the BmNPV in silkworms, and conducting a metabonomic study of the midgut tissues could reveal the silkworm’s anti-BmNPV mechanisms. With the advent of the current era of big data, the research and analysis of genomes [10], transcriptomes [11], proteomes [12] and metabonomes [13] of highly resistant and sensitive strains of silkworms will facilitate the analysis of antiviral mechanisms in silkworms. Recently, many investigators have integrated metabonomics and transcriptomics to study metabolic pathways, disease resistance, and stress resistance mechanisms of organisms under threat [14,15]. For instance, Xu et al. [16] conducted a metabonomic study on the metabolite differences between two silkworm varieties using gas chromatography–liquid chromatography–mass spectrometry. They concluded that the hemolymph in silkworms fed an artificial diet was vitamin deficient, but it was exceedingly rich in nitrogen-based metabolites such as urea and uric acid. Aside from the disturbance in amino acid metabolism, the silkworms’ poor intake of artificial feed was also caused by a simultaneous metabolic decline of carbohydrates, energy, and lipids.

For this study, we selected two silkworm varieties; Baiyu as the control group and Baiyu N as the high-BmNPV resistance group. The two silkworm varieties share a similar genetic makeup but have different BmNPV-resistance levels. After adding a high-concentration of BmNPV into our system, we used GC–MS metabonomics and RNA-seq to study how BmNPV changes the silkworms’ midgut tissues and endogenous metabolites, and to identify differentially expressed genes in the two silkworm varieties in order to define the stress response and BmNPV resistance mechanism.

## 2. Results

### 2.1. Inter-Sample Atlas Detection

A visual inspection of the samples was conducted using their respective total ion current (TIC) chromatograms, and the inspection revealed that the instrumental analysis atlas of all samples was characterized by high signal intensity, large peak capacity, long retention time, and good repeatability. As shown in Supporting File Figure 1 (Figure S1), when the same silkworm variety received different treatments, the inter-sample chromatographic peak difference was small. However, when different silkworm varieties received an identical treatment, the chromatographic peak difference was significant. The chromatographic peak difference among the different silkworm varieties was greater than that of silkworm varieties receiving different treatments. Supporting File Figure 1 shows a total of 1279 detected chromatographic peaks with a signal-to-noise ratio greater than 50 and 451 metabolites.

### 2.2. OPLS-DA Analysis

**Figure 1:**
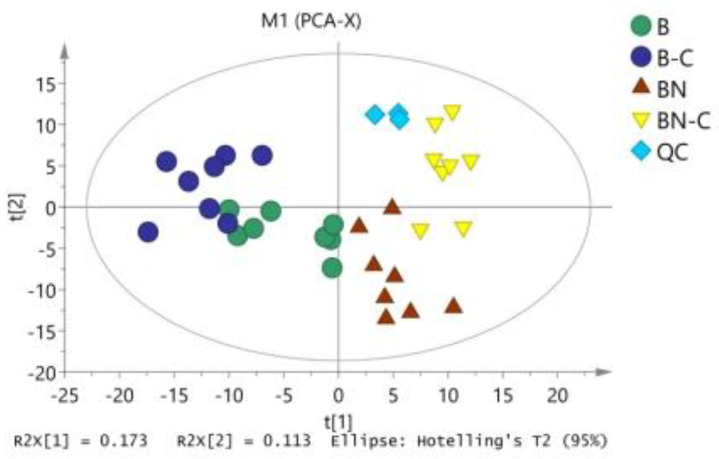
PCA analysis of midgut metabolites in four group samples. B is the Baiyu treatment group, B-C is the Baiyu control group, BN is the BaiyuN treatment group, BN-C is the BaiyuN control group.

The raw data were exported after data alignment, deconvolution, and normalization. We then used SIMCA 14.0 to conduct PCA on Baiyu and Baiyu N varieties before and after BmNPV induction (Figure 1) and found no apparent separation between the two silkworm varieties. The model fit the parameter [17,18] R2X (cum) that was obtained during PCA, which was equal to 0.553, thereby clarifying why the three major model components could explain 55.3% of variable X. Q2 (cum) was equal to 0.221, suggesting that the model had low predictive ability and further analysis was required.

**Figure 2:**
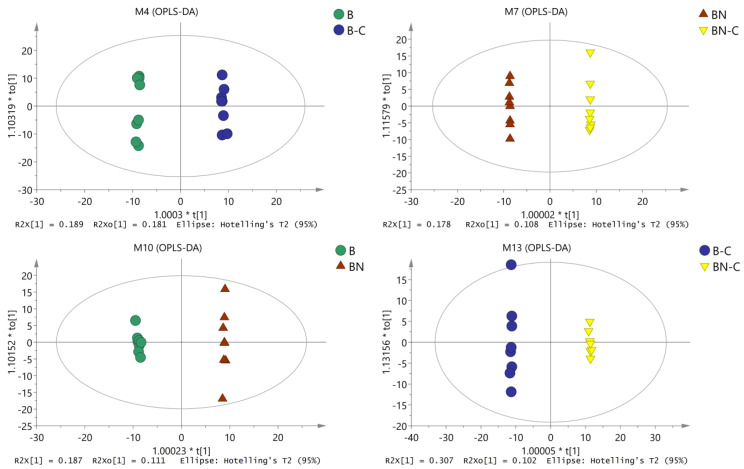
OPLS-DA analysis chart of midgut metabolites among the different samples.

We conducted OPLS-DA again to eliminate noise unrelated to the classifications (such as in the control and test groups) and to obtain more reliable metabolite information for the significant differences among the reference samples. Through this second OPLS-DA, we obtained inter-sample OPLS-DA-related parameters (Table 1) and analytic charts (Figure 2). As shown in Table 1 and Figure 2, OPLS-DA was conducted on the pairwise-compared samples, which further improved the model parameters. There was an obvious trend of separation and aggregation between midgut tissue metabolites of the BmNPV-sensitive Baiyu silkworms and the highly BmNPV-resistant Baiyu N silkworms before and after BmNPV induction. These findings suggest that the OPLS-DA model could explain and predict differences among the sample groups.

### 2.3. Screening of Differential Metabolites

#### 2.3.1. Metabonomic Comparison between Different Silkworm Varieties Pre- and Post BmNPV Infection

We combined single-dimensional and multidimensional analyses for screening differential metabolites between the highly BmNPV-resistant Baiyu N variety and the BmNPV-sensitive Baiyu variety. We compared the midgut metabolites of the two silkworm varieties before adding BmNPV, for example, BN-C vs. B-C. We then used the VIP value as the screening criterion and singled out 178 VIPs > 1.0, which presented a significant difference of *p* < 0.05 in the metabolites. Based on the different reference groups with different metabolite FC values, we obtained 101 metabolites in the high-resistant variety BN-C that had a concentration higher than that of the sensitive variety B-C, and 77 metabolites with a concentration lower than that of the sensitive variety B-C. For FC values, FC > 1 indicated an increase in the metabolite concentration and FC < 1 indicated a decrease in the metabolite concentration. Among the identified metabolites, the concentrations of 15 metabolites, including galactose, trehalose-6-phosphate, tetrahydrocorticosterone, and 2-hydroxyvaleric acid, in BN-C were more than two times those of B-C (2.01 to 72.68 times). A few metabolites appeared only in the highly BmNPV-resistant BN-C group, namely analyte 963, N-acetyl-5-hydroxytryptamine, 1-hexadecanol, and 2-amino-2-norbornanecarboxylic acid. The concentrations of 39 metabolites, including hippuric acid, 2-mercaptoethanesulfonic acid, and DL-*p*-hydroxyphenyllactic acid, in the highly BmNPV-resistant variety BN-C, were more than two times lower than those of the sensitive variety B-C. For these metabolites, the BN-C group’s metabolite concentration was only 0.02 to 0.48 times as high as the disease-sensitive variety B-C. Additionally, seven metabolites only appeared in the sensitive variety B-C, including 4-hydroxybenzyl cyanide, 1,3-cyclohexanedione, leucine, indolelactate, benzylsuccinic acid, phenylacetic acid, and farnesal (for details, see Supplementary Table S1).

Similarly, we compared the midgut metabolites of the highly BmNPV-resistant Baiyu N variety (BN) and BmNPV-sensitive Baiyu variety (B) after adding BmNPV, which is represented as BN vs. B. Using the VIP value as the screening criterion, we singled out 117 VIPs > 1.0 that showed a significant difference of *p* <0.05 in metabolites. We found 75 metabolites in BN with a concentration higher than B (FC >1) and 42 metabolites lower than B (FC < 1). We found the concentrations of eight metabolites in BN to be more than two times higher than those in B, including analyte 1153, 6-hydroxycaproic acid, d-galacturonic acid, glucuronic acid, sorbitol, alanine, D-erythro-sphingosine, and pyrophosphate. The concentrations of these metabolites ranged from 2.11 to 13.56 times higher in BN than those in B. Among these metabolites, 4-hydroxymandelonitrile was specific to BN. Moreover, there were seven metabolites in BN with concentrations more than two times lower than those in B, including N-carbamylglutamate, N-methylaniline, 3-aminoisobutyric acid, cortexolone, analyte 1371, L-kynurenine, and acetylsalicylic acid. The concentrations of these metabolites were 0.12 to 0.48 times as high as B. Additionally, the metabolites benzoylformic acid, 6-deoxy-D-glucose, and octanal were specific to B (for details, see Supplementary Table S2).

#### 2.3.2. Metabonomic Comparison between the Same Silkworm Variety before and after Adding BmNPV 

A metabonomic comparison between the highly BmNPV-resistant variety Baiyu N before adding BmNPV (BN) and after adding BmNPV (BN-C) was conducted and represented as BN vs. BN-C. Again, we used the VIP value as the screening criterion and singled out 117 VIPs >1.0 with a significant difference of *p* < 0.05. Using this method, we identified 55 metabolites with increased concentrations after adding BmNPV, including 29 metabolites with concentrations increased by more than two times (2.05 to 10.06 times). Meanwhile, the expression of differentially the metabolites 2,3-pyridinedicarboxylic acid, 6-hydroxycaproic acid, N-(3-aminopropyl)morpholine, methylmalonic acid, L-4-hydroxyphenylglycine, and N-ethylglycine changed significantly and increased more than 7-fold. In addition, phenylacetic acid, indolelactate, 1,3-cyclohexanedione, and analyte 173 were specific to BN. The concentrations of 62 metabolites decreased in the BN-C group. By contrast, the same metabolite concentrations were 0.077 to 0.93 times as high as BN. The concentrations of 11 metabolites decreased by more than two times in BN, including 2-amino-2-norbornanecarboxylic acid (BCH), N-acetylisatin, urea, and N-acetyl-5-hydroxytryptamine. Moreover, the metabolites 1-hexadecanol and octanal were specific to BN-C (for details, see Supplementary Table S3).

The same type of metabonomic comparison was also performed on the BmNPV-sensitive Baiyu variety before adding BmNPV (B) and after adding BmNPV (B-C), represented as B vs. B-C. Using the VIP value as the screening criterion, we singled out 124 VIPs > 1.0, and there was a significant difference of *p* < 0.05 in the metabolites. The concentrations of 73 metabolites increased after adding BmNPV, including ten metabolite concentrations that increased by more than two times (2.03 to 59.69 times). In addition, three metabolites were specific to B, namely adipamide, benzoylformic acid, and 2-amino-2-norbornanecarboxylic acid. Moreover, the concentrations of 51 metabolites decreased in the B-C group. Among these, the concentrations of 12 metabolites decreased significantly by more than two times with concentrations only 0.052 to 0.48 times as high as B. Only one metabolite, 2,4-diaminobutyric acid, was specific to B-C (for details, see Supplementary Table S4).

Table 2 is based on the FC values of different metabolites in the reference groups. As shown in Table 2, there were metabolite changes in both Baiyu N (BN) and Baiyu (B) before and after adding BmNPV. After adding BmNPV, the concentrations of 33 metabolites in Baiyu N significantly increased (FC > 2). Moreover, most of these metabolites were related to energy metabolism. However, after adding BmNPV, the concentrations of only 13 metabolites in Baiyu significantly increased. As such, there were more significant metabolite concentration changes in the disease-resistant Baiyu N variety than in the BmNPV-sensitive Baiyu variety. These metabolite fluctuations and changes might be correlated with the silkworms’ BmNPV resistance.

We also sorted peculiar metabolites in the midgut tissues of Baiyu N and Baiyu varieties, as shown in Table 3. During pairwise comparison, 25 metabolites, including 2-amino-2-norbornanecarboxylic acid, benzoylformic acid, and adipamide, were detected in only one sample. We speculated these metabolites might be correlated with the two varieties’ difference in the BmNPV resistance level. From the VIP values of these peculiar metabolites, most of the metabolite VIP values were not high (given *p* < 0.01 in peculiar metabilities, we used the VIP values for screening differential metabolites). In other words, during pairwise comparisons, these peculiar metabolites were not necessarily the most important factors in the disease resistance differences between silkworm varieties.

Therefore, it was necessary to identify the reason for the large BmNPV-resistance difference between Baiyu N and Baiyu through the analysis of the differential metabolites’ metabolic pathways.

### 2.4. Differential Metabolites Metabolic Pathways Analysis

We acquired the Kyoto Encyclopedia of Genes and Genomes ID (KEGG ID) for most differential metabolites (some had no KEGG ID) through the ID conversion function on the MBRole (http://csbg.cnb.csic.es/mbrole/) website. We then analyzed the metabolic pathways of the differential metabolites and related enzymes. One metabolite is often involved in multiple metabolic pathways, and each pathway involves many metabolites. To facilitate the interpretation of results, we performed enrichment analysis of the metabolic pathways of all metabolites and generated a metabolic pathways enrichment map for the reference samples (Supplementary Tables S5–S8). The differential metabolites of B vs. B-C were involved in 44 metabolic pathways, whereas the differential metabolites of BN vs. B were involved in 51 metabolic pathways. The differential metabolites of BN vs. BN-C were involved in 36 metabolic pathways, whereas the differential metabolites of BN-C vs. B-C were involved in 54 metabolic pathways.

**Figure 3:**
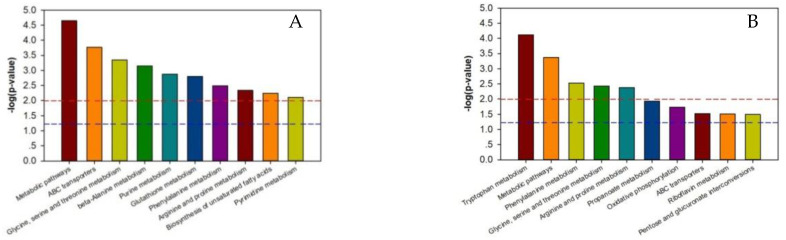

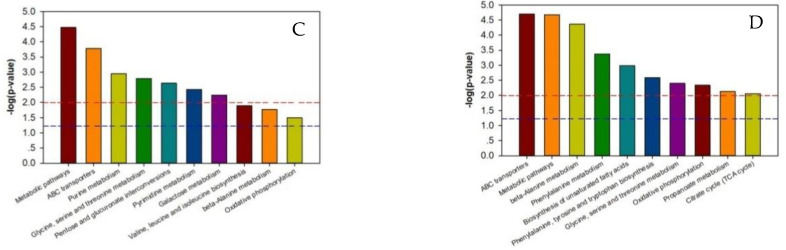
Enrichment map of metabolic pathways (top ten *p*-value) among test sample groups. Note: The dotted red line indicates a *p*-value of 0.01, while the dotted blue line indicates a *p*-value of 0.05. When the metabolite bars exceed the dotted red line or blue line, the corresponding signal pathways are significant. (**A**):B vs. BC; (**B**) BN vs. BNC; (**C**) BN vs. B; (**D**) BNC vs. BC. B is the Baiyu treatment group, B-C is the Baiyu control group, BN is the BaiyuN treatment group, BN-C is the BaiyuN control group.

As shown in Supplementary Tables S5–S8, the *p*-value of each metabolic pathway was different from those of the other pathways, indicating that there were differences between the metabolic pathways. The lower the metabolic pathway *p*-value, the greater the metabolic pathway difference among the reference groups. The greater differences indicated a higher chance that the metabolic pathway was involved in regulating the two silkworm varieties’ disease resistance, thereby causing a different response to BmPNV. We enriched the top ten metabolic pathways with regard to the *p*-value and generated an enrichment graph of the different test group’s metabolic pathways. We used the metabolic pathway as the horizontal ordinate coordinate and -log2 (*p*-value) as the vertical coordinate. As shown in Figure 3, in B vs. B-C, there were ten highly contributive metabolic pathways, including ABC transporters, glycine, beta-alanine metabolism, serine, and threonine metabolism. In BN vs. BN-C, there were ten highly contributive metabolic pathways, and the metabolism of the following metabolites: tryptophan, phenylalanine, glycine, serine, threonine, arginine, and proline. Because of Baiyu and Baiyu N’s significant difference in the BmNPV resistance level, with Baiyu N showing 1000 times more BmNPV resistance in LC_50_ calculation starting from the second instar, the ten metabolites involved in regulating Baiyu’s BmNPV resistance may be unrelated to Baiyu N’s high resistance and may not play key roles. Therefore, when analyzing the metabolic pathways with important roles in regulating Baiyu N’s BmNPV resistance, we excluded the five metabolic pathways that appeared in both B vs. B-C and BN vs. BN-C, including ABC transporters, and the metabolism of the following metabolites: glycine, serine, threonine, phenylalanine, arginine, and proline. We paid special attention to the remaining metabolic pathways, including oxidative phosphorylation, pentose and glucuronate interconversions, and the metabolism of tryptophan, propanoate, and riboflavin.

### 2.5. Analysis and Mining of Transcriptome Data

#### 2.5.1. Analysis of Differentially Expressed Genes

DESeq was used to analyze the transcriptome data of the differentially expressed genes among the reference samples using the following screening criteria: FC ≥ 2 and FDR < 0.01. Table 4 shows the differentially expressed genes among the reference samples.

In general, an insect kills or inhibits an invading pathogen by phagocytosis, autophagy, encapsulation, nodulation, apoptosis and RNA interference. The insect must mobilize many genes to fight the invading pathogen. Species with weak disease resistance need to muster more energy and resources to defend themselves against pathogens in order to survive and continue their lineage. By contrast, species with strong disease resistance may only need to activate a few key factors to achieve a strong antagonistic effect. As a result, when the differentially expressed genes were analyzed, the number of differentially expressed genes detected in the more BmNPV-sensitive Baiyu B vs. B-C was much greater than that in the BmNPV-resistant BN vs. BN-C group (Table 4).

#### 2.5.2. KEGG Annotation of Differentially Expressed Genes

**Figure 4:**
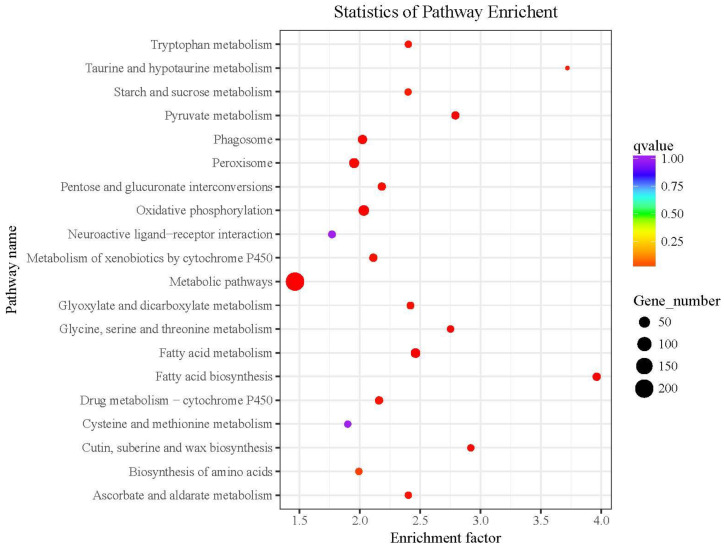
KEGG cluster map of differentially expressed genes detected by RNA-seq. The red boxes indicate the pathways we focus on.

The differentially expressed genes in the KEGG pathway enrichment analysis and the 20 pathways with the lowest significant Q values are shown in Figure 4. The level of KEGG enrichment was measured by the enrichment factor, q-value, and the number of the genes enriched on the relevant pathway. The larger the enrichment factor, the higher the enrichment level. The q-value is the *p*-value corrected by multiple hypothesis testing with a range from 0 to 1. The closer the q-value is to 0, the higher the enrichment level.

**Figure 5:**
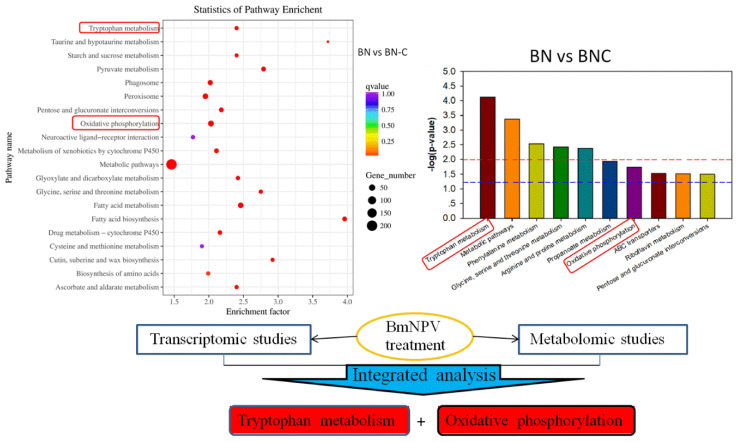
Integrated analysis of transcriptomic and metabolomic studies. The red boxes show overlapping results from both two –omics analyses.

We found that tryptophan metabolism and oxidative phosphorylation might be two of the five metabolic pathways related to Baiyu and Baiyu N silkworms’ different BmNPV-resistant levels. These pathways were also detected by RNA-seq, as shown in the red box in Figure 5. Thus, we have reason to believe that tryptophan metabolism and oxidative phosphorylation are closely related to the different BmNPV resistance between the two silkworm varieties in this study.

## 3. Discussion

In general, silkworms have an innate immune system similar to that of vertebrates. The system resists invading microorganisms in four stages, namely identification, regulation, signal transduction, and response [19]. For example, Siglec is a molecule that might play a role in the pattern recognition of silkworms’ BmNPV resistance, whereas the high expression of two silkworm proteases [20], Bmserpin-5 trypsin-like serine protease and chymotrypsin inhibitor (CI-8A), in a silkworm variety infected with BmNPV, has been shown to be an important regulator of innate immunity [21]. The confirmed BmNPV-resistant effectors in silkworms include Bm NADH-oxidoreductase-like (NOX) [22], Bmlipase-1 [23], Bm SP-2 [24] and multiform RFPs [25]. The three main signal transduction pathways involved in the virus response in insects are Toll, Imd, and Jak-STAT [26].

However, existing studies show different silkworm varieties respond to BmNPV differently and have diverse resistant mechanisms. This study showed that tryptophan metabolism and oxidative phosphorylation are closely related to silkworms’ BmNPV-resistance.

Studies have reported intra-intestinal tryptophan metabolism and the conversion of several molecules by intestinal bacteria, such as IAid, IAA, IPA, and IAAid, all of which are ligands of AhR [27]. The AhR signal is considered to be a key barrier immunoreaction component. It is critical for the renewal of intestinal epithelial cells and the integrity of the intestinal mucosal barrier. It can act on many types of immunocytes, including intra-epithelial lymphocytes, Th17 cells, macrophages, and dendritic cells. Moreover, AhR is directly activated by dietary molecules and xenobiotics [27]. After silkworms were fed a high-concentration of BmNPV in this study, AhR was activated under the virus infection, which in turn acted on many different types of immunocytes. This mechanism stimulated the silkworm’s immune system to resist BmNPV infection and resulted in BmNPV resistance.

Oxidative phosphorylation is an important biochemical process in cells and the main step for creating “energy currency” during the generation of ATP. There are two types of oxidative phosphorylation, one is metabolite-linked and the other is respiratory chain-linked. Approximately, 95% of an organism’s ATP comes from respiratory chain-linked oxidative phosphorylation. For example, ATP is generated in the respiratory chain during electron transfer [28]. After silkworms were attacked by a high-concentration of BmNPV, they had to muster all of their energy to protect themselves from BmNPV infection; therefore, in this case, energy metabolism is particularly important. The metabolomic results in this paper show that the main differential metabolites in Baiyu N and Baiyu silkworm varieties (BN vs. B) included saccharides, amino acids, amines, alcohols, and glycosides. These metabolites are small molecules directly involved in energy metabolism. Moreover, the number of differential metabolites was higher in BN vs. BN-C than that in B vs. B-C. In other words, Baiyu N variety silkworms have more ATP and are more BmNPV resistant because they have more energy resources to fight infection compared to Baiyu variety silkworms.

## 4. Materials and Methods

### 4.1. Test Materials and Sample Preparation

The control group Baiyu is highly sensitive to BmNPV, whereas the test group Baiyu N is highly resistant to BmNPV. The construction of Baiyu N was conducted as previous reported [4]. Briefly, a batch of silkworm sample of Baiyu strain was tested for susceptibility to BmNPV. The silkworm variety N carrying BmNPV resistance genes and showing the highest resistance to BmNPV was selected and used for further experiments. Baiyu was chosen as the recipient and silkworm variety N was selected as the donor to introduce the disease resistance genes into the recipient variety through hybridization technology. Then, the recipient variety Baiyu was used as recurrent parents to carry out four rounds of backcrossing to purify and fix the disease resistance genes. After subsequent selection to improve and stabilize the economic characters of backcross generations, the silkworm variety BaiyuN with high resistance to BmNPV infection was obtained. As a result, the LC_50_ value of BaiyuN to BmNPV virus was up to 1 × 10^9^ polyhedra/mL, a thousand-fold increase compared to the conventional Baiyu strain.

The two silkworm groups were reared on the same kind of mulberry leaf using the same method until the larvae hatched at the fifth instar. We hatched 200 larvae for each silkworm variety and selected 100 of these larvae as the control group. The remaining 100 larvae were fed 1.0 × 10^9^/mL of BmNPV solution at a dose of 8 µL per larva. After 12 h, midgut tissues were harvested from the silkworms of different groups and immediately stored at −80 °C for later use. In this study, B represented the Baiyu virus-infected group, B-C represented the Baiyu control group, BN represented the Baiyu N virus-infected group, and BN-C represented the Baiyu N control group. For each silkworm group, we used gas chromatography–mass spectrometry (GC–MS) metabolomic analysis for half of the samples and RNA sequencing (RNA-seq) for the remaining samples.

### 4.2. Sample Preprocessing

The silkworm samples stored at −80 °C were processed as quality control samples by grinding, internal marking, vortex oscillation, submerging in an ice-water bath, ultrasound extraction, and centrifugation. The samples were then freeze dried, oximated, and mixed with a derivating agent before applying GC–MS metabolomic analysis and RNA-seq. 

GC-MS analysis was conducted with a 7890-5977A GC/MSD (Agilent Technologies, Inc., CA, USA). The chromatographic conditions followed the methods of Li et al. [13] as follows: DB-5MS capillary column (30 m × 0.25 mm × 0.25 um, Agilent J&W Science, Folsom, CA, USA); carrier gas was high purity helium (purity not less than 99.999%); flow rate was 1.0 mL/min; and temperature of the inlet was 260 °C. The injection volume was 1 μL not diverted and the solvent was delayed for 5 min. For programmed heating, the initial temperature of the column temperature box was 60 °C, and the programmed temperature was 8 °C /min to 125 °C, 5 °C /min to 210 °C, 10 °C /min to 270 °C, and 20 °C /min to 305 °C for 5 min. The mass spectrometry conditions were as follows: electron bombardment ion source (EI), ion source temperature 230 °C, fourth-stage rod temperature 150 °C, and electron energy 70 eV. Scanning mode was full scan mode (SCAN), with quality scanning range of m/z 50 to 500. A quality control (QC) sample was inserted into every 16 analysis samples to examine the repeatability of the whole analysis process.

A total amount of 1 μg RNA per sample was used as input material for the RNA sample preparations. Sequencing libraries were generated using NEBNext UltraTM RNA Library Prep Kit for Illumina (NEB, Ipswich, MA, USA) following the manufacturer’s recommendations and index codes were added to attribute sequences to each sample. The clustering of the index-coded samples was performed on a cBot Cluster Generation System using TruSeq PE Cluster Kit v4-cBot-HS (Illumia) according to the manufacturer’s instructions. After cluster generation, the library preparations were sequenced on an Illumina Hiseq 2000 platform and paired-end reads were generated.

All metabolomic analysis and RNA-seq was performed by Shanghai Luming Bio-Technology Limited (shanghai, China).

### 4.3. GC-MS Analysis

After the raw GC–MS data (in D format) were converted into a general format (CDF) through ChemStation software (version E.02.02.1431, Agilent, CA, USA), they were imported into ChromaTOF software (version 4.34, LECO, St Joseph, MI) for preprocessing. The NIST and Fiehn databases were then used for qualitative analysis of metabolites, and peak alignment was performed to obtain the 3D data matrix in CSV format.

Following log2 (*p*-value) conversion, the 3D data matrix values were imported into SIMCA software (version 14.0, Umetrics, Umeå, Sweden). Principal component analysis (PCA) was first conducted to observe the samples’ overall distribution and the stability of the entire analytic process. Thereafter, orthogonal partial least squares discriminant analysis (OPLS-DA) was conducted to reveal the differences in the overall metabolic profiles among the silkworm reference groups, and an OPLS-DA model and OPLS-DA analysis chart were obtained for each sample.

### 4.4. Screening Differential Metabolites

We combined multidimensional and single-dimensional analyses to identify differential metabolites in each silkworm reference group using a variable importance in the projection (VIP) value >1 as the principal criterion of the OPLS-DA model and a *p*-value < 0.05 for the t-test (Student’s t-test). The differential metabolite ratio among the reference groups was calculated based on the average differential metabolite content of eight duplicate samples in each group. For example, the fold change (FC) value was expressed as the ratio of metabolite expression quantities between the two samples. (For instance, the FC value of B vs. B-C = Average (B)/Average (B-C). The FC values for the remaining reference samples were calculated in the same way). For convenience, when a certain metabolite value was equal to 0, it was expressed as 0.000001.

### 4.5. Analysis of Metabolic Pathways of Differential Metabolites

The Kyoto Encyclopedia of Genes and Genomes ID (KEGG ID) [14] of each differential metabolite was acquired through the ID conversion function at the MBRole website (http://csbg.cnb.csic.es/mbrole/). We then analyzed the metabolic pathways of the differential metabolites and related enzymes. The KEGG IDs of the differential metabolites were used for pathway enrichment analysis to obtain the metabolic pathway enrichment results [15]. The difference was significant when *p* < 0.05. Lower *p*-values resulted in larger differences in metabolic pathways among the reference groups.

### 4.6. Analysis and Screening of Differentially Expressed Genes

The differentially expressed genes were analyzed using DESeq software. We used a FC ≥2 and a false-discovery rate (FDR) <0.01 as the criteria for screening differentially expressed genes. To prevent false positives in the identified differentially expressed genes, significant *p*-values were corrected using the Benjamini–Hochberg correction method. We used the FDR as a key indicator for screening differentially expressed genes to obtain a set of differentially expressed genes between samples [29,30].

### 4.7. KEGG Annotation of Differentially Expressed Genes

We added KEGG annotations to differentially expressed genes. The major metabolic pathways and signal transduction pathways were then identified for the differentially expressed genes based on significant pathway enrichment. Finally, the pathways in KEGG were classified by function [31].

## Figures and Tables

**Table 1:** Inter-sample PCA and OPLS-DA analysis-related parameters.

No.	Model	Type	A	*N*	R2X(cum)	R2Y(cum)	Q2(cum)	R2	Q2
All	M1	PCA-X	6	35	0.553		0.221		
B vs. B-C	M2	PCA-X	3	16	0.51		0.145		
BN vs. BN-C	M5	PCA-X	3	16	0.488		0.0336		
BN vs. B	M8	PCA-X	4	16	0.586		0.0885		
BN-C vs. B-C	M11	PCA-X	4	16	0.653		0.208		
B vs. B-C	M4	OPLS-DA	1+2+0	16	0.426	0.999	0.894	0.978	−0.216
BN vs. BN-C	M7	OPLS-DA	1+3+0	16	0.461	1	0.835	0.742	−1.080
BN vs. B	M10	OPLS-DA	1+2+0	16	0.409	0.999	0.854	0.904	−0.196
BN-C vs. B-C	M13	OPLS-DA	1+2+0	16	0.499	1	0.966	0.813	−0.471

B is the Baiyu treatment group, B-C is the Baiyu control group, BN is the BaiyuN treatment group, BN-C is the BaiyuN control group.

**Table 2:** Metabolites of different silkworm varieties midgut tissues.

Tissue	Group	Metabolites	Up	Down	QuantMass	RT(min)	VIP	*p*-Value	FC
Midgut	B vs. B-C	124	73	51	56–451	5.1286–35.1984	1.0065–2.1926	2.4055 × 10^−8^–0.0494	7.9322 × 10^−6^–310452.02
	BN vs. BN-C	117	56	61	58–451	5.1766–36.1096	1.0555–2.3677	9.5441 × 10^−8^–0.0499	2.6607 × 10^−6^–685403.10
	BN vs. B	117	75	42	71–468	5.1955–34.9107	1.0277–2.2455	3.4469 × 10^−10^–0.0484	4.3374 × 10^−6^–58324.76
	BN-C vs. B-C	178	101	77	56–397	5.2509–38.0193	1.0014–1.8053	3.1763 × 10^−12^–0.0499	3.5462 × 10^−7^–596800.06

Note: The variable importance in the projection (VIP) value is obtained from the OPLS-DA model. The higher the VIP value, the greater the variable’s contribution to grouping. The *p*-value is the t-test result and is used to evaluate whether there is a significant variable difference between two groups of samples, with *p* < 0.05 indicating a significant difference and *p* < 0.01 indicating a very significant difference. The FC is the ratio of the average expression quantity of metabolites in two sample groups. When FC > 1 it represents up-regulation, while FC < 1 represents down-regulation. If the expression quantity of a metabolite is 0, it is expressed as 0.000001 for calculation convenience.

**Table 3:** Specific metabolites from midgut tissues of different silkworms.

Tissue	Group	Metabolites	QuantMass	RT(min)	VIP	*p* Value	FC	Average(B)	Average(BC)
Midgut	B vs. B-C	2-Amino-2-norbornanecarboxylic acid	89	8.3346	1.5773	0.0044	51086.82	0.051087	0.000001
		Benzoylformic acid	255	34.9107	1.5502	0.0045	67933.12	0.067933	0.000001
		Adipamide	215	25.6626	1.5467	0.0045	310452.02	0.310452	0.000001
		2,4-diaminobutyric acid	176	14.0483	2.0223	1.22 × 10^−5^	7.93 × 10^−6^	0.000001	0.126068
	BN vs. BN-C	Phenylacetic acid	83	5.2971	2.3675	1.31 × 10^−11^	685403.1	0.685403	0.000001
		Indolelactate	202	34.8854	1.5517	0.0436	37819.53	0.037820	0.000001
		1,3-Cyclohexanedione	216	13.7762	1.5538	0.0204	25094.28	0.025094	0.000001
		Analyte 173	327	7.7791	1.5601	0.01957	12816.88	0.012817	0.000001
		octanal	96	22.5501	2.3677	9.54 × 10^−14^	2.66 × 10^−6^	0.000001	0.375837
		1-Hexadecanol	184	10.4910	2.0596	0.0008	5.33 × 10^−6^	0.000001	0.187692
	BN vs. B	4-Hydroxymandelonitrile	205	25.4390	1.5148	0.0362	58324.76	0.058325	0.000001
		octanal	96	22.5501	2.0407	1.07 × 10^−5^	4.34 × 10^−6^	0.000001	0.230551
		6-deoxy-D-glucose	156	19.7094	1.7947	0.001	6.80 × 10^−6^	0.000001	0.147048
		Benzoylformic acid	255	34.9107	1.5556	0.0045	1.47 × 10^−5^	0.000001	0.067933
	BN-C vs. B-C	2-Amino-2-norbornanecarboxylic acid	89	8.3346	1.8053	3.18 × 10^−12^	88556.14	0.088556	0.000001
		1-Hexadecanol	184	10.4910	1.5828	0.0008	187691.72	0.187692	0.000001
		N-Acetyl-5-hydroxytryptamine	231	8.0323	1.3072	0.0077	495947.17	0.495947	0.000001
		Analyte 963	72	24.1345	1.0548	0.0205	596800.07	0.596800	0.000001
		Phenylacetic acid	83	5.2971	1.8047	4.94 × 10^−9^	1.02 × 10^−6^	0.000001	0.981171
		Farnesal	217	27.0758	1.7906	1.21 × 10^−5^	3.55 × 10^−7^	0.000001	2.819911
		leucine	158	5.9749	1.5922	0.0001	1.52 × 10^−5^	0.000001	0.065595
		Benzylsuccinic acid	131	12.3266	1.4033	0.0006	3.93 × 10^−6^	0.000001	0.254367
		1,3-Cyclohexanedione	216	13.7762	1.3965	0.0008	2.72 × 10^−5^	0.000001	0.036744
		4-Hydroxybenzyl cyanide	221	10.1969	1.0416	0.0226	3.22 × 10^−5^	0.000001	0.031097
		Indolelactate	202	34.8854	1.0462	0.0234	1.46 × 10^−5^	0.000001	0.068349

**Table 4:** Number of differentially expressed genes among samples.

DEG Set	DEG Number	Up-Regulated	Down-Regulated	KEGG Pathway
B vs. B-C	2651	1406	1245	561
BN vs. B	2622	1577	1045	460
BN-C vs. B-C	1522	1023	499	276
BN vs. BN-C	434	209	225	96

Note: DEG Set is a differentially expressed gene set name. The DEG Number is the number of differentially expressed genes. Up-regulated here is the number of up-regulated genes, while down-regulated here is the number of down-regulated genes.

## Supplementary Materials

Supplementary materials can be found at https://www.mdpi.com/1422-0067/21/13/4707/s1.
