# Supplementary figures and images for: Metabolic Characterisation of the Midgut of Bombyx mori Varieties after BmNPV Infection Using GC-MS-Based Metabolite Profiling
Source: Int J Mol Sci. 2020 Jul 1;21(13):4707. doi: 10.3390/ijms21134707 (PMC7369710; doi:10.3390/ijms21134707)

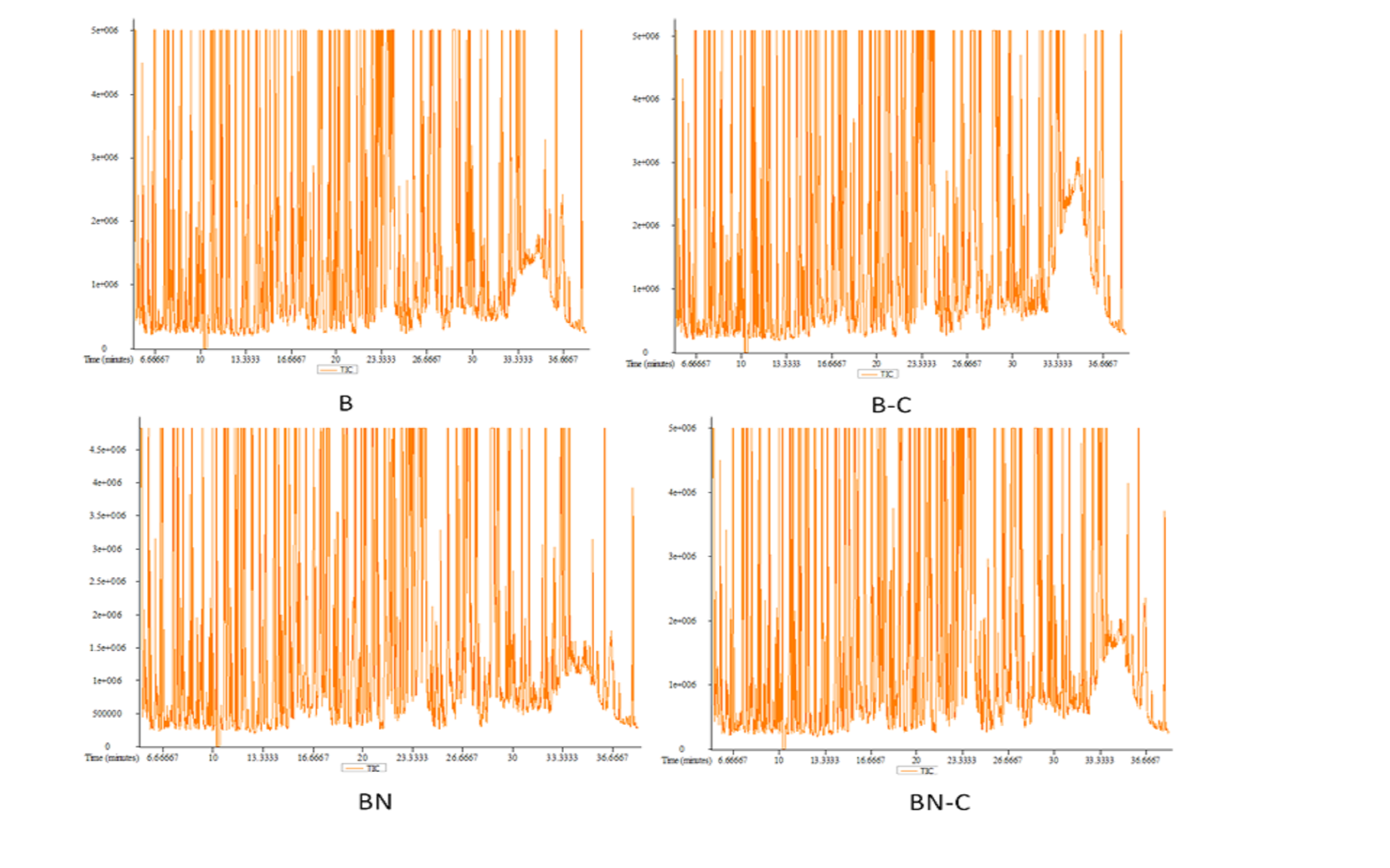

Supplement: Supplementary file 1 [file ijms-21-04707-s001.zip › Figure S1.tif]
